# Multi-trait multi-environment models in the genetic selection of segregating soybean progeny

**DOI:** 10.1371/journal.pone.0215315

**Published:** 2019-04-18

**Authors:** Leonardo Volpato, Rodrigo Silva Alves, Paulo Eduardo Teodoro, Marcos Deon Vilela de Resende, Moysés Nascimento, Ana Carolina Campana Nascimento, Willian Hytalo Ludke, Felipe Lopes da Silva, Aluízio Borém

**Affiliations:** 1 Federal University of Viçosa—Department of Plant Science, University Campus, Viçosa, Minas Gerais, Brazil; 2 Federal University of Viçosa—Department of General Biology, University Campus, Viçosa, Minas Gerais, Brazil; 3 Federal University of Mato Grosso do Sul—Department of Plant Science, University Campus, Chapadão do Sul, Mato Grosso do Sul, Brazil; 4 Federal University of Viçosa—Department of Statistics, University Campus, Viçosa, Minas Gerais, Brazil; College of Agricultural Sciences, UNITED STATES

## Abstract

At present, single-trait best linear unbiased prediction (BLUP) is the standard method for genetic selection in soybean. However, when genetic selection is performed based on two or more genetically correlated traits and these are analyzed individually, selection bias may arise. Under these conditions, considering the correlation structure between the evaluated traits may provide more-accurate genetic estimates for the evaluated parameters, even under environmental influences. The present study was thus developed to examine the efficiency and applicability of multi-trait multi-environment (MTME) models by the residual maximum likelihood (REML/BLUP) and Bayesian approaches in the genetic selection of segregating soybean progeny. The study involved data pertaining to 203 soybean *F*_2:4_ progeny assessed in two environments for the following traits: number of days to maturity (DM), 100-seed weight (SW), and average seed yield per plot (SY). Variance components and genetic and non-genetic parameters were estimated via the REML/BLUP and Bayesian methods. The variance components estimated and the breeding values and genetic gains predicted with selection through the Bayesian procedure were similar to those obtained by REML/BLUP. The frequentist and Bayesian MTME models provided higher estimates of broad-sense heritability per plot (or heritability of total effects of progeny; $h_{prog}^{2}$) and mean accuracy of progeny than their respective single-trait versions. Bayesian analysis provided the credibility intervals for the estimates of $h_{prog}^{2}$. Therefore, MTME led to greater predicted gains from selection. On this basis, this procedure can be efficiently applied in the genetic selection of segregating soybean progeny.

## Introduction

Soybean [*Glycine max* (L.) Merrill] is the fourth most widely grown crop in the world. This species is originally from China and is the major crop in the USA, Brazil, Argentina, and many other countries [1]. Soybean is currently grown from low to high latitudes, where it is used as a source of oil, protein, biodiesel, etc. [2]. In this scenario, because the genotype × environment (G×E) interaction plays an essential role in genotypic expression, it must be considered in the evaluation and selection of superior genotypes [3–5].

The selection of segregating soybean progeny is a rather complex process because the traits of agronomic importance (e.g., maturity, yield, etc.) are of quantitative nature. Furthermore, because some traits are correlated with each other, selection based on one of them leads to alterations in others [6,7]. In soybean breeding, most of the available methods for the selection of progeny or lines are useful in the analysis of a single trait measured either in a single environment [8,9] or in various environments with the incorporation of the G×E interaction [10–12]. However, researchers often face situations in which multiple traits are measured across multiple environments [13]. Moreover, selection bias may arise when genetic selection is performed based on two or more genetically correlated traits (due to pleiotropism, imbalance in the gametic phase, and/or the common influence of the environment) and these are analyzed individually. This is especially true in sequential selection [13–15].

To reduce selection bias, Henderson *et al*. [16] proposed the multiple-trait BLUP method. An additional advantage of jointly modeling multiple traits compared to analyzing each trait separately is that the inference process appropriately accounts for the correlation between the traits, which helps to increase prediction accuracy, statistical power, and parameter estimation accuracy [14,16,17]. Despite the improvements provided by the multiple-trait BLUP (Best Linear Unbiased Prediction) method, to the best of our knowledge, there are few studies combining multi-trait models under a multi-environment approach.

The multiple-trait BLUP procedure is ideal, as it makes it possible to simultaneously analyze traits that are correlated with each other and that exhibit covariance heterogeneity across experimented environments [18,19]. In this way, a covariance structure is applied to each random factor in the model; e.g., the progeny effects, the G×E interaction effects, and the residual effects [20,21]. Even though data collected in plant breeding studies often present a multi-trait multi-environment structure, as they take into consideration the genetic correlations and the G × E interaction, more complex models are required, rendering the computation process more laborious. Some studies have demonstrated the potential of the Bayesian approach for genetic evaluation in plant breeding considering multi-trait or multi-environment models [22–25]. In this approach, the parameters are interpreted as random variables, following the law of probability, which assumes a priori knowledge [25,26].

In view of the above-described situation, the present study proposes to examine the efficiency and applicability of multi-trait multi-environment (MTME) models in the selection of segregating soybean progeny, using phenotypic data, by the frequentist (FMTME) and Bayesian (BMTME) methodologies. Effective biometric tools were exploited and compared with the usual tools employed in genetic breeding aiming at increasing the predicted genetic gain. In this way, study demonstrated it was possible to attain these goals by selecting soybean genotypes with better genetic potentials (desirable phenotypic traits) in different evaluation environments.

## Material and methods

### Experimental data

Three populations (Pop) belonging to the Soybean Breeding Program at the Federal University of Viçosa (UFV) were obtained from crosses between divergent inbred lines (Pop1: TMG123RR/M7211RR; Pop2: UFVSCitrinoRR/UFVSTurquezaRR; and Pop3: M7908RR/M7211RR). These lines were classified into different relative maturity groups according to the soybean crop management classification [27], aiming to exploit genetic variability for the selection of productive progeny. Seventy-two, 71, and 60 *F*_2_ plants of populations 1, 2, and 3, respectively, were separately bulk-harvested and threshed. One sample was collected from each *F*_2:3_ progeny to compose the 203 *F*_2:4_ progeny that were used in this study by the within-progeny bulk method [28,29].

To evaluate these progeny, two trials were conducted in the 2016/2017 crop year at the Teaching, Research, and Academic-Extension Units at UFV. One of them took place in Capinópolis—MG, Brazil (18°40'48" S latitude, 49°33'58" W longitude; 530 m altitude) and the other in Viçosa—MG, Brazil (20º45'45" S latitude, 42º49'27" W longitude; 647 m altitude).

The experiments were set up as a randomized complete block design with three replicates per site. Plots consisted of two 3.0-m rows spaced 0.5 m apart, with a plant stand density of 13 seeds per meter, totaling a density of 256,000 plants ha^–1^. All plant management operations were undertaken in accordance with the requirements of the crop in the region [30].

Three target agronomic traits were evaluated, namely: number of days to maturity (DM, days), 100-seed weight (SW, g), and average seed yield per plot (SY, g). The first variable, DM, corresponds to the number of days before 95% of the pods were mature, as indicated by their color [31]. To evaluate the SW and SY traits, the grains were dried to 13% moisture. All data and code to run the main models used in this study are available in S1 Table and S2 Table, respectively.

### Frequentist statistical analyses

The Restricted Maximum Likelihood/Best Linear Unbiased Prediction (REML/BLUP) procedure was adopted for statistical analyses under a frequentist approach (Patterson and Thompson [32] and Henderson [33]). The frequentist single-trait multi-environment (FSTME) statistical model associated with the evaluation of segregating progeny in a randomized block design in two environments, with one observation per plot, is given by:

y=Xb+Zg+Wi+e,

where ***y*** is the vector of phenotypes; ***b*** is the vector of block effects added to the overall mean (assumed fixed); ***g*** is the vector of progeny effects (assumed random), in which $g∼N(0,\sigma_{g}^{2})$; ***i*** is the vector of the G×E interaction effects (random), in which $i∼N(0,\sigma_{\mathrm{int}}^{2})$; and ***e*** is the vector of residuals (random), in which $e∼N(0,\sigma_{e}^{2})$. The capital letters (**X**, **Z** and **W**) represent the incidence matrices for the effects of ***b***, ***g*,** and ***i***, respectively. The ***b*** vector encompasses all replicates of all locations. In this case, this vector comprises the effects of locations and of replicates within locations. The goodness of fit was obtained using Akaike information criteria (AIC [34]), defined by AIC = −2*LogL*+2*p*, in which *LogL* is the log-likelihood function and *p* is the number of estimated parameters, according to Little *et al*. [35]; and the Likelihood Ratio Test (LRT), following Wilks [36], using Chi-square Statistics with one degree of freedom, which is calculated by the following equation: *λ* = 2[*Log*_*e*_*L*_*p*+1_−*Log*_*e*_*L*_*p*_], in which *L*_*p*+1_ and *L*_*p*_ are the maximum likelihood associated with the full and the reduced models, respectively.

In the frequentist multi-trait multi-environment (FMTME) approach, this model was transformed into ***g***~***N***(0,∑_***g***_⊗***I***), ***i***~***N***(0,∑_int_⊗***I***) and ***e***~***N***(0,∑_***e***_⊗***I***), where ∑_*g*_ is the progeny covariance matrix; ∑_int_ is the G×E interaction covariance matrix; ∑_*e*_ is the residual covariance matrix; *I* is an identity matrix with order appropriate to the respective random effect; and ⊗ denotes the Kronecker product. ∑_*g*_, ∑_int_, and ∑_*e*_ are also unstructured covariance structures (US) [18,19]. The following ***Y***_*1*_, ***Y***_*2*_, and ***Y***_*3*_ are the vectors of observed responses for each trait (DM, SW, and SY) and (co)variance matrix estimates:

$$Y=\left[\begin{array}{c} Y_{1}\\ \cdots \\ Y_{2}\\ \cdots \\ \vdots \\ Y_{3} \end{array}\right];\;\sum {}_{g}=\left[\begin{array}{ccc} \sigma_{g1}^{2} & \sigma_{g1g2}^{2} & \sigma_{g1g3}^{2}\\ \sigma_{g2g1}^{2} & \sigma_{g2}^{2} & \sigma_{g2g3}^{2}\\ \sigma_{g3g1}^{2} & \sigma_{g3g2}^{2} & \sigma_{g3}^{2} \end{array}\right];\;\sum {}_{\mathrm{int}}=\left[\begin{array}{ccc} \sigma_{i1}^{2} & \sigma_{i1i2}^{2} & \sigma_{i1i3}^{2}\\ \sigma_{i2i1}^{2} & \sigma_{i2}^{2} & \sigma_{i2i3}^{2}\\ \sigma_{i3i1}^{2} & \sigma_{i3i2}^{2} & \sigma_{i3}^{2} \end{array}\right]\;and\;\sum {}_{e}=\left[\begin{array}{ccc} \sigma_{e1}^{2} & \sigma_{e1e2}^{2} & \sigma_{e1e3}^{2}\\ \sigma_{e2e1}^{2} & \sigma_{e2}^{2} & \sigma_{e2e3}^{2}\\ \sigma_{e3e1}^{2} & \sigma_{e3e2}^{2} & \sigma_{e3}^{2} \end{array}\right]$$

To perform the statistical analyses of the FSTME and FMTME models and obtain the variance components and breeding values, we applied the ASReml 4.1 package [21] of integrated R software (Development Core Team—[37]).

### Bayesian statistical analyses

The single-trait multi-environment and multi-trait multi-environment models as well as the covariance matrix structures (US) of the frequentist analyses were used according to the Bayesian approach (BSTME and BMTME, respectively) via Markov Chain Monte Carlo (MCMC) to estimate the variance components and genetic parameter. To gather further information about representation of model, matrix, and vectors structures as well as priori probability distributions, we recommend reading Mrode [18] and Gilmour *et al*. [21] and recent publications Junqueira *et al*. [23], Torres *et al*. [25] and Mora and Serra [38].

We assumed that the variance-covariance matrices follow an Inverse-Wishart (IW) distribution and independent Inverse-Gamma (IG) distributions, which were used as a priori to model the variance-covariance matrix [39–41]. The ensuing covariance matrix distribution is such that all standard-deviation parameters have Half-t distributions and the correlation parameters have uniform distributions on (-1,1) for a particular choice of the IW shape parameter [13]. The advantage of this approach is that it allows us to choose the shape and scale parameters that achieve high non-arbitrary information of all standard deviations and correlation parameters [40].

The full (considering the genotype and G × E interaction effects) models were compared with the reduced (disregarding the genotype or G × E interaction effects) models by the deviance information criterion (DIC) proposed by Spiegelhalter *et al*. [42]: $DIC=D(\bar{\theta })+2p_{D}$, where $D(\bar{\theta })$ is a point estimate of the deviance obtained by replacing the parameters with their posterior mean estimates in the likelihood function and *p*_*D*_ is the effective number of parameters in the model. Models with lower DIC should be preferred over models with higher DIC.

The Bayesian models (BSTME and BMTME) were implemented in the “MCMCglmm” package [43,44] of R software (R Development Core Team—[37]). A total of 1,000,000 samples were generated, and assuming a burn-in period and sampling interval of 500,000 and 5 iterations, respectively, this resulted in a final total of 100,000 samples. The convergence of MCMC was checked by the criterion of Geweke [45], which was performed using the “boa” [46] and “CODA” (Convergence Diagnosis and Output Analysis) [47] R packages.

### Genetic and non-genetic components

Broad-sense heritability per plot (or heritability of total effects from progeny) for the frequentist and Bayesian models were computed based on the approximated estimators, as discussed in Piepho *et al*. [48], using the following expression:

$$h_{prog}^{2}=\frac{\sigma_{g}^{2}}{\sigma_{g}^{2}+\frac{\sigma_{\mathrm{int}}^{2}}{n}+\frac{\sigma_{e}^{2}}{nr}}$$

where $\sigma_{g}^{2}$: variance of progeny; $\sigma_{\mathrm{int}}^{2}$: variance of the progeny × environment interaction; $\sigma_{e}^{2}$: error variance; *n*: number of locations; and *r*: number of replicates. For the Bayesian models, the posterior estimates were calculated from the posterior samples of the variance components obtained by the model.

The accuracy of progeny selection for the frequentist models (FSTME and FMTME) was estimated based on the following expression [49]:

$$r_{\hat{g}g}=\sqrt{(\sigma_{g}^{2}-PEV)/\sigma_{g}^{2}},$$

where $\sigma_{g}^{2}$ is the genotypic variance and *PEV* is the prediction error variance extracted from the diagonal of the generalized inverse of the coefficient matrix of the mixed-model equations.

For the Bayesian approach (BSTME and BMTME models), the accuracy of progeny selection was estimated according to Resende *et al*. [19] from the posterior distribution, given by:

$${\tilde{r}}_{\hat{g}g}=[1-s(\tilde{g})/\tilde{g}],$$

where $s(\tilde{g})$ is the standard deviation of the predicted breeding value. According to Resende *et al*. [19], in Bayesian inference, the variance of the very parameter that is assumed as a random variable is computed.

The “boa” [46] and “bayesplot” [50,51] packages of R software were used to calculate and plot the highest posterior density (HPD) intervals for all parameters, respectively. Estimates of the coefficient of experimental variation (*CVe*) and selective accuracy ($r_{\hat{g}g}$) were used to evaluate the experimental quality of the models [52].

### Genetic correlation

To determine the genetic covariance by the frequentists and Bayesian single-trait models (FSTME and BSTME, respectively), a pairwise analysis of the sum of phenotypic values of the traits was performed. Thus, the covariances were obtained, as proposed by Resende *et al*. [53], using the following expression:

$$\sigma_{g(trait_{i},trait_{j})}=\frac{\sigma_{g}^{2}{}_{\left(trait_{i}+trait_{j}\right)}-\sigma_{g(trait_{i})}^{2}-\sigma_{g(trait_{j})}^{2}}{2},$$

where $\sigma_{g}^{2}{}_{\left(trait_{i}+trait_{j}\right)}$ is the variance of the sum of phenotypic values of traits *i* and *j*; $\sigma_{g(trait_{i})}^{2}$ is the genotypic variance of trait *i*; and $\sigma_{g(trait_{j})}^{2}$ is the genotypic variance of trait *j*. Genetic covariances by the multi-trait multi-environment models (FMTME and BMTME models) were obtained directly by the mixed-model output from each applied methodology.

The genetic correlation coefficients between the DM, SW, and SY traits were obtained, as suggested by Piepho *et al*. [54], using the expression below for all models:

$$\rho_{\left(trait_{i},trait_{j}\right)}=\frac{\sigma_{g(trait_{i},trait_{j})}}{\sqrt{\sigma_{g(trait_{i})}^{2}\sigma_{g(trait_{j})}^{2}}}$$

### Progeny selection

The Spearman rank correlation coefficient was calculated between the BLUP (breeding value) of the progeny from the analyses of FSTME, FMTME, BSTME, and BMTME, and its significance was verified using nonparametric bootstrap in the R package ‘boot’ [55,56]. The agreement between the selected progeny was also checked by the coincidence index (*CI*) proposed by Hamblin and Zimmermann [57], as shown below:

$$CI=\frac{\left(A-C\right)}{\left(M-C\right)},$$

where *A* is the number of coincident progeny in the two methods, *M* is the number of selected progeny, and *C* is the number of progeny coincident due to chance (*C* = *bM*, where *b* is the selection intensity = 0.15; i.e., 15%).

Selection gain was predicted for each trait (DM, SW, and SY) based on the expression below:

$$SG=\frac{\sum_{i=1}^{n}GV_{i}}{n},$$

where *GV*_*i*_ is the predicted genotypic value of progeny *i* and *n* is the number of selected progeny (30).

In order to perform a simultaneous selection and infer about the efficiency of selection gain for each evaluated trait between the frequentist (FSTME and FMTME) and Bayesian (BSTME and BMTME) models, we applied the additive genetic index using Selegen REML/BLUP software (AGI–[58]). The predicted breeding values of the selected progeny were thus compared by the frequentist and Bayesian approaches. The weights for each trait were defined based on the coefficients of genetic variation [59]. For all traits, the progeny were selected to increase the phenotypic expression, or provide the highest BLUP. After the direction of selection was defined, the genotypic values for each progeny [weighted by the pre-established weights (*CV*_*g*_) for each trait] were summed, generating the AGI value. Subsequently, they were organized in descending order.

## Results

### Analysis of deviance and model fitting

The significance of progeny (G) and G×E interaction effects of the FSTME model were evaluated. Significant G and G×E interaction effects (P ≤ 0.01) were detected in the LRT for the DM, SW, and SY traits (Table 1). According to the AIC from the results obtained with the FSTME model, the model including the G and G×E interaction effects (full model) showed the best fit (lowest AIC value) for all traits (Table 2). Thus, according to the two methodologies, the full FSTME model was the most suitable to estimate the genetic parameters and predict the genotypic values. Likewise, the FMTME model was also appropriate, as it showed the lowest AIC value. In the Bayesian models, all chains achieved convergence by the criterion of Geweke [45]. Overall, the DIC values were smaller when using the full Model (considering genotype and G × E interaction effects), being the difference in relation to full Model higher than 2 (Table 2), which according to Spiegelhalter *et al*. [42] it’s enough to suggest that the use of full Model can lead to higher accuracy in estimating the parameters (Table 2). Therefore, since this model component is important the “best” genotypes measured in different environments couldn’t the same.

**Table 1:** Deviance and likelihood ratio test (LRT) for number of days to maturity (DM), 100-seed weight (SW) (grams), and average seed yield per plot (SY) (grams) evaluated in 203 soybean *F*_2:4_ progeny for single-trait multi-environment (FSTME) frequentist analysis.

Effect	DM		SW		SY	
	Deviance	LRT	Deviance	LRT	Deviance	LRT
Progeny (G)	7048.74	141.17**	2398.04	94.15**	5837.97	15.68**
G×E interaction	7010.14	102.57**	2362.57	58.68**	5853.38	31.09**
Full model	6907.57		2303.89		5822.29	

** Significant at the 0.01 probability level according to the chi-square test.

**Table 2:** Akaike information criteria for the full model and deviance information criteria for the full (considering genotype and G × E interaction effects) and reduced [disregarding the genotype (−*prog*) and interaction (−int) effects] models for number of days to maturity (DM), 100-seed weight (SW) (grams), and average seed yield per plot (SY) (grams) via frequentist and Bayesian single-trait multi-environment (FSTME and BSTME) and multi-trait multi-environment (FMTME and BMTME) models.

		Akaike information criteria (AIC)		
Model	Trait	Full model	Reduced model1 (−*prog*)	Reduced model2 (−int)
FMTME	DM, SW, SY	14861.22	15184.53	15051.87
FSTME	DM	6913.569	7052.743	7014.143
FSTME	SW	2309.892	2402.042	2366.568
FSTME	SY	5828.291	5841.969	5857.377
		Deviance information criteria (DIC)		
BMTME	DM, SW, SY	20577.3	20694.71	21019.89
BSTME	DM	8700.467	8741.243	8938.965
BSTME	SW	4157.547	4198.951	4303.262
BSTME	SY	7890.409	7900.89	7968.122

### Genetic and non-genetic components

The estimates of genotypic variance $\left(\sigma_{g}^{2}\right)$ and G×E interaction variance $\left(\sigma_{\mathrm{int}}^{2}\right)$ were higher and lower, respectively, when obtained via FMTME and BMTME, for all traits. By contrast, the residual variance ($\sigma_{res}^{2}$) and individual phenotypic variance ($\sigma_{phen}^{2}$) estimates were similar regardless of the procedure used for the frequentist models, whereas in the Bayesian models these estimates were higher for all traits, even for the genotypic component $\left(\sigma_{g}^{2}\right)$. However, the $\sigma_{res}^{2}$ and $\sigma_{phen}^{2}$ estimates were similar between the BSTME and BMTME models. As a consequence, both the frequentist and Bayesian MTME models led to higher estimates of $h_{prog}^{2}$, *CV*_*g*_, and *Ac*_*prog*_ compared with the frequentists and Bayesian single-trait models. The exception was the SW trait, for which no significant differences were observed (Table 3) between single- and multi-trait multi-environment models. For the other components, both procedures led to results proportional to the estimates of $\sigma_{g}^{2}$ and $\sigma_{\mathrm{int}}^{2}$.

**Table 3:** Estimates of variance components and genetic and non-genetic parameters for number of days to maturity (DM), 100-seed weight (SW) (grams), and average seed yield per plot (SY) (grams) evaluated in 203 soybean *F*_2:4_ progeny via frequentist single-trait multi-environment (FSTME) and multi-trait multi-environment (FMTME) and Bayesian single-trait multi-environment (BSTME) and multi-trait multi-environment (BMTME) models.

Component	FSTME			FMTME			BSTME			BMTME		
	DM	SW	SY	DM	SW	SY	DM	SW	SY	DM	SW	SY
$\sigma_{g}^{2}$	128.625	1.8045	8.6171	130.947	1.8084	10.4882	129.598	1.8158	8.3801	131.616	1.8161	11.0459
$\sigma_{\mathrm{int}}^{2}$	33.9228	0.6244	10.0727	31.6741	0.6228	8.2995	34.2204	0.6278	10.2834	31.2917	0.6253	6.9344
$\sigma_{res}^{2}$	56.5253	1.5008	36.4566	56.5234	1.5006	36.4419	56.7499	1.5081	36.6665	57.1089	1.5152	37.5989
$\sigma_{phen}^{2}$	219.073	3.9297	55.1464	219.144	3.9318	55.2297	220.569	3.9518	55.3299	220.017	3.9566	55.5792
$h_{prog}^{2}$	0.8298	0.7624	0.4368	0.8383	0.7631	0.5064	Table 4^a^	Table 4 ^a^	Table 4 ^a^	Table 4 ^a^	Table 4 ^a^	Table 4^a^
$C_{\mathrm{int}}^{2}$	0.1548	0.1589	0.1827	0.1445	0.1584	0.1503	0.1552	0.1588	0.1859	0.1422	0.158	0.1248
*CV*_*g*_	0.0791	0.0729	0.141	0.0798	0.073	0.1555	0.0794	0.0732	0.1390	0.0801	0.0732	0.1596
*CV*_*e*_	0.0524	0.0665	0.2901	0.0524	0.0665	0.2899	0.0525	0.0666	0.2908	0.0527	0.0668	0.2945
*Ac*_*prog*_	0.9087	0.8691	0.6557	0.9485	0.8713	0.933	0.9633	0.6337	0.7367	0.9733	0.6355	0.8941

$\sigma_{g}^{2}$: genotypic variance; $\sigma_{\mathrm{int}}^{2}$: genotype × environment interaction variance; $\sigma_{res}^{2}$: residual variance; $\sigma_{phen}^{2}$: individual phenotypic variance; $h_{prog}^{2}$: mean broad-sense heritability per plot; $C_{\mathrm{int}}^{2}$: coefficient of determination of the genotype × environment interaction effects; *CV*_*g*_: genotypic coefficient of variation; *CV*_*e*_: residual coefficient variation; and *Ac*_*prog*_: mean accuracy of progeny.

^a^Results showed in Table 4.

Compared with FSTME, the FMTME model provided increases of the orders of 1.81, 0.22, and 21.72% in the estimate of $\sigma_{g}^{2}$ and reductions of 6.63, 0.26, and 17.61% in the estimate of $\sigma_{\mathrm{int}}^{2}$ for the respective traits DM, SW, and SY. As a result, the $h_{prog}^{2}$ estimates increased by 1.03, 0.1, and 15.94%; *CV*_*g*_, by 0.88, 0.14 and 10.28%; and *Ac*_*prog*_, by 4.38, 0.25, and 42.29% for the respective traits.

Compared with BSTME, the BMTME model led to 1.56, 0.02, and 31.81% higher estimates of $\sigma_{g}^{2}$ and 8.56, 0.4, and 32.57% lower estimates of $\sigma_{\mathrm{int}}^{2}$ for the respective traits DM, SW, and SY. The reduced ranging of $h_{prog}^{2}$ (lower and upper difference) with Bayesian MTME credible intervals (probability of 95%) were 9.66, 0.22, and 42.44% for the DM, SW, and SY traits, respectively (Table 4). Thus, the mean increased by 1.08, 0.00, and 24.01%; *CV*_*g*_ increased by 0.88, 0.14, and 10.82%; and *Ac*_*prog*_ increased by 1.04, 0.28, and 21.37% for the respective traits.

**Table 4:** Posterior inferences for mode, mean, median, and higher posterior density (HPD) interval of the broad-sense heritability per plot, considering the proposed Bayesian single-trait multi-environment (BSTME) and multi-trait multi-environment (BMTME) models for number of days to maturity (DM), 100-seed weight (SW) (grams), and average seed yield per plot (SY) (grams).

	BSTME					BMTME				
Trait	$h_{prog}^{2}$			HPD (95%)		$h_{prog}^{2}$			HPD (95%)	
	Mode	Mean	Median	Lower	Upper	Mode	Mean	Median	Lower	Upper
DM	0.8354	0.8282	0.8298	0.7798	0.874	0.842	0.8375	0.8388	0.7936	0.8787
SW	0.765	0.76	0.7628	0.6905	0.8247	0.775	0.765	0.7628	0.6908	0.8247
SY	0.44	0.4204	0.4282	0.2406	0.586	0.5361	0.5292	0.531	0.4304	0.6292

**Fig 1:**
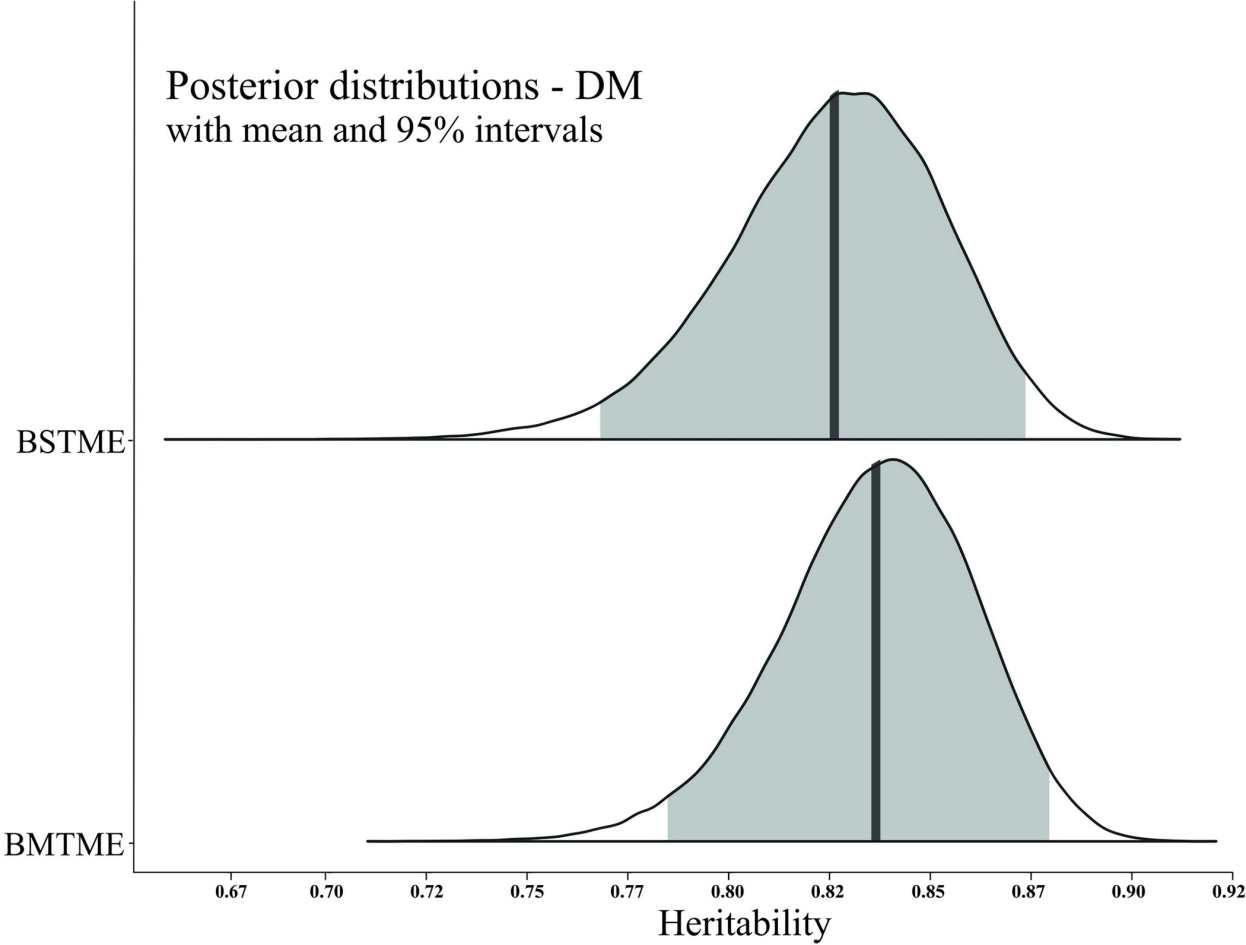
Posterior density for the Bayesian single-trait multi-environment (BSTME) (top) and multi-trait multi-environment (BMTME) (bottom) models of the broad-sense heritability per plot for number of days to maturity (DM). The solid color represents the posterior density of 95% intervals and the solid vertical line indicates the mean.

**Fig 2:**
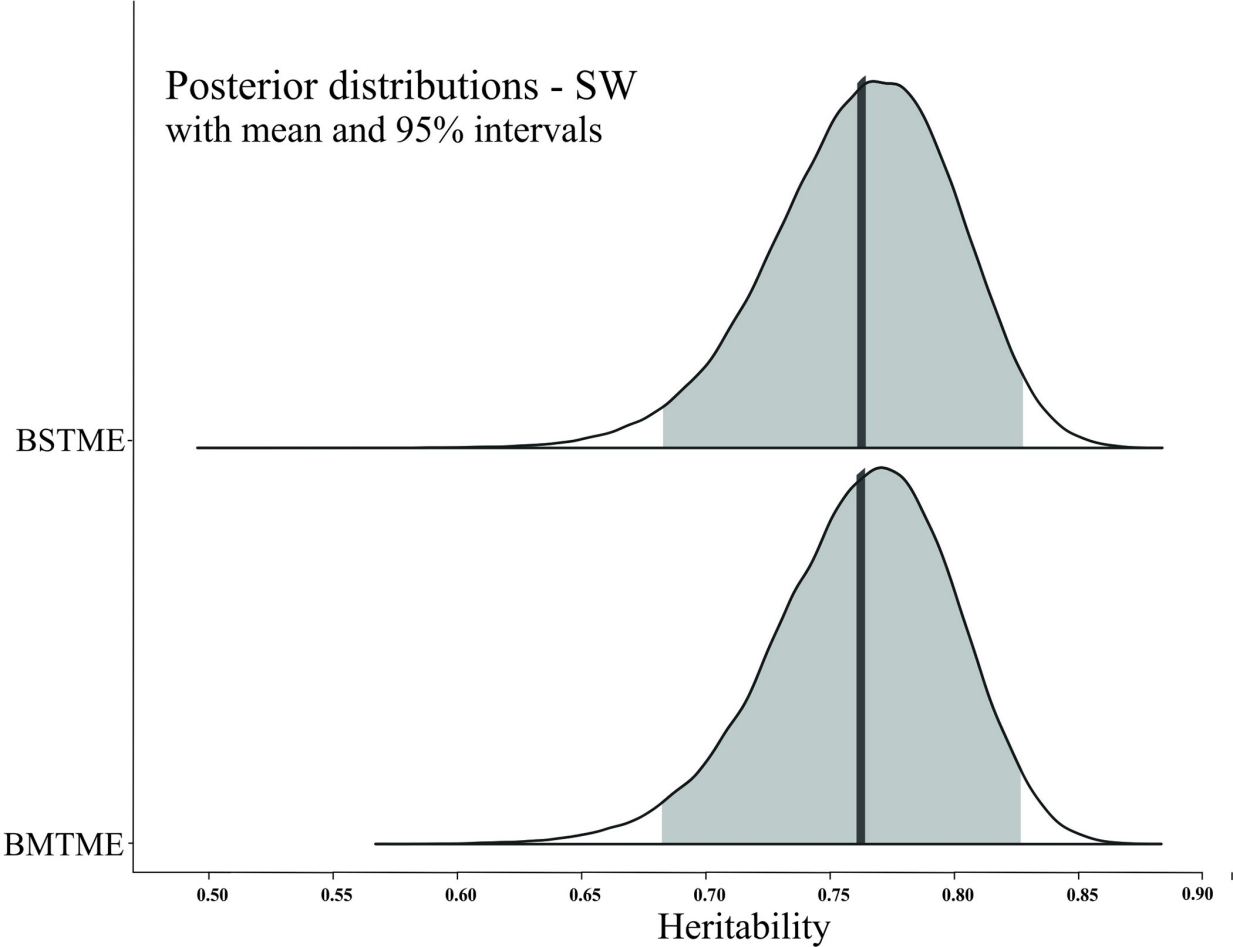
Posterior density for the Bayesian single-trait multi-environment (BSTME) (top) and multi-trait multi-environment (BMTME) (bottom) models of the broad-sense heritability per plot for 100-seed weight (SW; grams). The solid color represents the posterior density of 95% intervals and the solid vertical line indicates the mean.

**Fig 3:**
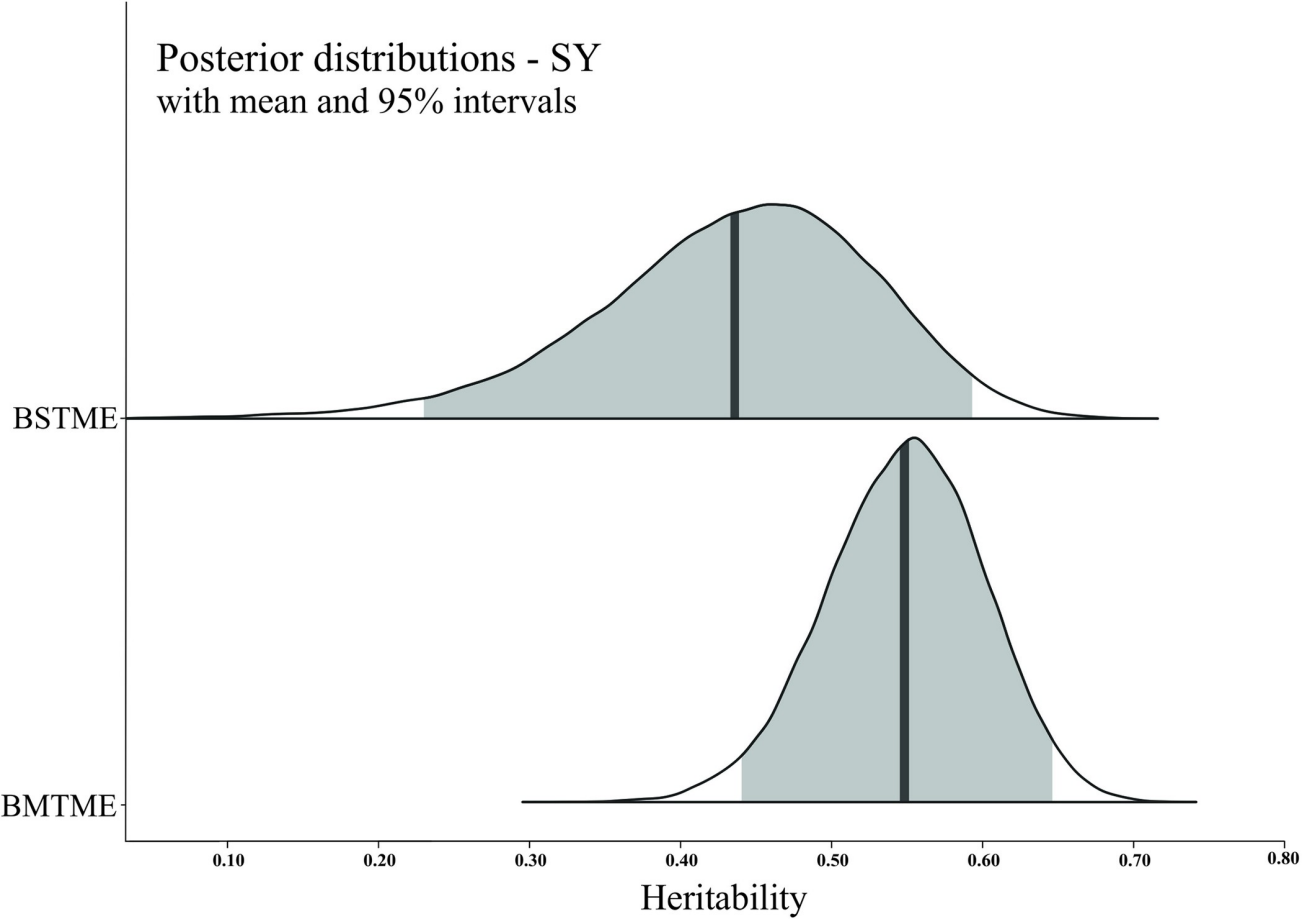
Posterior density for the Bayesian single-trait multi-environment (BSTME) (top) and multi-trait multi-environment (BMTME) (bottom) models of the broad-sense heritability per plot for average seed yield per plot (SY; grams). The solid color represents the posterior density of 95% intervals and the solid vertical line indicates the mean.

The posterior density intervals of heritability genetic parameters (Table 4, and Figs 1–3) were accessed to assist in the selection of genotypes in the Bayesian models. Thus, the breeding values and their HPD intervals obtained from the Bayesian STME and MTME models for each trait can be useful tools in progeny selection. Posterior density intervals of estimates of genotypic variance for BSTME and BMTME were showed in S1–S4 Figs.

### Genetic correlation

Genetic correlations between the DM, SW, and SY traits obtained by the frequentist and Bayesian models are given in Table 5. For the DM-SW and SW-SY pairs, low correlations were detected in every comparison, indicating absence of linear associations. However, a high and positive correlation was found between DM and SY in both methodologies. Additionally, the correlation between this pair of traits estimated via FSTME and BSTME exceeded the parameter space. The same was not true when it was estimated via FMTME and BMTME, which showed similar values within the parameter space and were thus more realistic (not biased).

**Table 5:** Genetic correlations between number of days to maturity (DM), 100-seed weight (SW) (grams), and average seed yield per plot (SY) (grams) evaluated in 203 soybean *F*_2:4_ progeny via frequentist single-trait multi-environment (FSTME) and multi-trait multi-environment (FMTME) and Bayesian single-trait multi-environment (BSTME) and multi-trait multi-environment (BMTME) models.

Correlation	FSTME	FMTME	BSTME	BMTME
*ρ* (DM,SW)	–0.1090	–0.1107	–0.1048	–0.1078
*ρ* (DM,SY)	^¶^	0.9718	^¶^	0.9724
*ρ* (SW,SY)	–0.0153	–0.0217	–0.0164	–0.0296

¶: value higher than unity

### Progeny selection

The Spearman rank correlations between breeding values via single- and multi-trait multi-environment models (FSTME and FMTME; BSTME and BMTME) were significant in all comparisons. This correlation was medium for SY (79.16 and 70.92) in the frequentist and Bayesian models, respectively, and high for the other comparisons. These results were confirmed by the agreement (Table 6).

**Table 6:** Predicted selection gain, agreement, and Spearman rank correlation in the selection of the 30 best soybean progeny via frequentist (FSTME and FMTME) and Bayesian (BSTME and BMTME) models for number of days to maturity (DM), 100-seed weight (SW; grams), and average seed yield per plot (SY; grams) evaluated in 203 soybean *F*_2:4_ progeny.

Trait	Predicted selection gain				Agreement (%)	
	FSTME	FMTME	BSTME	BMTME	FSTME × FMTME	BSTME × BMTME
DM	12.70	13.22	12.67	13.32	93.30 (0.9349^b^)	93.31 (0.9289)
SW	1.92	1.93	1.92	1.92	100.00 (0.9963)	100.00 (0.9965)
SY	3.11	3.74	2.99	3.84	46.40 (0.7916)	46.60 (0.7992)

^b^ Spearman rank correlation

In the frequentist and Bayesian analyses, the predicted selection gains were equivalent for all traits. However, the MTME models showed greater gains. For the SW trait, both the frequentist and the Bayesian procedures generated very similar results (Table 6). For DM and SY, however, there was less agreement between the selected progeny, especially for the SY trait, which culminated in greater discrepancy between the gains predicted from selection.

For the DM trait, the MTME models led to increased gains predicted from selection: 4.01 and 5.13% for the frequentist and Bayesian methodologies, respectively. Considering the SY trait, for which the agreement between the selected progeny was lower than 50%, the MTME models showed increases of 20.26% and 28.43% (frequentist and Bayesian models, respectively) in the gain predicted from selection, as compared with their respective single-trait models (Table 6).

When the AGI was used for the simultaneous selection of the 30 best soybean progeny, higher index gains were found for the FMTME and BMTME models (Tables 7 and 8) compared with the gain of the overall mean of AGI. The gains predicted from selection were similar for both models (FMTME and BMTME), for all traits. Moreover, greater gains were observed for the SY variable (15.03 and 15.71% for FMTME and BMTME, respectively).

**Table 7:** Order, progeny (Prog), breeding value (u+g), and Additive Genetic Index (AGI) of the 30 progeny selected simultaneously via frequentist single-trait multi-environment (FSTME) and multi-trait multi-environment (FMTME) models for number of days to maturity (DM), 100-seed weight (SW; grams), and average seed yield per plot (SY; grams) evaluated in 203 soybean *F*_2:4_ progeny.

		FSTME					FMTME			
Order	Prog	Trait			AGI	Prog	Trait			AGI
		DM	SW	SY			DM	SW	SY	
1	521	156.92	20.66	23.70	31.39	521	158.31	20.54	25.49	29.79
2	267	148.06	20.36	26.51	31.13	550	157.50	20.22	25.15	29.47
3	550	157.05	20.34	22.93	31.02	267	155.95	20.22	25.06	29.33
4	556	152.91	19.98	22.83	30.44	556	153.87	19.90	24.09	28.80
5	235	152.08	19.13	25.31	30.38	235	157.97	19.00	25.25	28.72
6	545	149.86	20.16	23.07	30.36	537	151.56	20.14	23.46	28.67
7	537	151.66	20.20	22.17	30.33	545	151.65	20.08	23.55	28.66
8	554	151.38	20.32	21.83	30.31	562	157.65	19.03	24.86	28.66
9	520	153.18	19.77	22.49	30.24	554	150.68	20.27	23.21	28.65
10	262	154.56	18.17	26.44	30.20	262	162.68	17.99	26.43	28.63
11	56	154.15	19.80	21.79	30.18	520	153.64	19.69	23.96	28.63
12	562	158.58	19.12	21.98	30.14	56	153.21	19.74	23.79	28.60
13	568	157.47	19.60	20.81	30.10	568	154.41	19.55	23.98	28.59
14	272	154.70	19.24	22.65	30.06	567	153.70	19.61	23.72	28.54
15	515	149.72	20.63	20.35	30.04	272	155.49	19.16	24.37	28.49
16	266	147.93	19.03	25.45	30.01	515	146.55	20.62	22.04	28.36
17	567	158.44	19.63	19.86	29.98	215	155.10	18.97	24.35	28.33
18	215	151.38	19.07	23.96	29.97	569	155.41	18.91	24.13	28.28
19	546	145.02	20.03	22.67	29.80	266	154.80	18.91	24.39	28.28
20	571	154.15	19.50	20.77	29.76	269	157.11	18.49	24.68	28.23
21	269	156.09	18.58	22.69	29.75	571	151.64	19.46	23.22	28.20
22	569	158.02	18.97	20.90	29.75	51	155.26	18.80	24.05	28.19
23	514	151.80	19.49	21.60	29.75	239	155.65	18.60	24.34	28.13
24	531	151.52	19.69	20.96	29.71	514	151.12	19.44	23.15	28.13
25	239	153.60	18.69	23.08	29.70	531	149.66	19.65	22.74	28.09
26	51	158.16	18.83	20.61	29.61	546	146.99	19.98	22.25	28.02
27	212	150.14	18.09	25.59	29.60	524	152.57	19.01	23.47	28.02
28	516	150.42	19.58	21.20	29.60	544	149.87	19.51	22.71	28.01
29	524	153.04	19.07	21.70	29.59	512	154.70	18.58	23.97	27.99
30	544	152.91	19.54	20.36	29.59	516	149.23	19.54	22.62	27.96
Mean of sel.		153.16	19.51	22.54	30.08		153.80	19.45	23.95	28.48
Overall mean		143.45	18.43	20.82	28.21		143.45	18.43	20.82	26.49
Gain (%)		6.77	5.85	8.27	6.64		7.21	5.55	15.03	7.50

**Table 8:** Order, progeny (Prog), breeding value (u+g), and Additive Genetic Index (AGI) of the 30 progeny selected simultaneously via Bayesian single-trait multi-environment (BSTME) and multi-trait multi-environment (BMTME) models for number of days to maturity (DM), 100-seed weight (SW; grams), and average seed yield per plot (SY; grams) evaluated in 203 soybean *F*_2:4_ progeny.

		BSTME					BMTME			
Order	Prog	Trait			AGI	Prog	Trait			AGI
		DM	SW	SY			DM	SW	SY	
1	521	156.86	20.65	23.60	31.23	521	158.49	20.53	25.58	29.96
2	267	148.06	20.37	26.28	30.95	550	157.61	20.22	25.23	29.64
3	550	157.02	20.34	22.85	30.86	267	156.48	20.22	25.23	29.50
4	556	152.90	19.98	22.75	30.28	556	154.00	19.90	24.16	28.96
5	235	152.08	19.13	25.14	30.21	235	158.30	19.01	25.41	28.90
6	545	149.87	20.16	22.99	30.20	537	151.64	20.13	23.49	28.83
7	537	151.62	20.19	22.12	30.17	562	157.59	19.03	24.92	28.82
8	554	151.38	20.32	21.80	30.16	545	151.83	20.08	23.62	28.82
9	520	153.16	19.77	22.42	30.09	262	163.15	18.01	26.68	28.81
10	56	154.13	19.81	21.74	30.03	554	150.73	20.26	23.24	28.80
11	262	154.55	18.18	26.24	30.03	520	153.73	19.69	24.01	28.79
12	562	158.54	19.12	21.94	29.98	56	153.22	19.74	23.82	28.75
13	568	157.48	19.60	20.81	29.95	568	154.28	19.54	23.99	28.75
14	272	154.68	19.25	22.58	29.90	567	153.49	19.60	23.70	28.70
15	515	149.69	20.63	20.37	29.90	272	155.53	19.16	24.43	28.65
16	567	158.42	19.63	19.89	29.84	515	146.49	20.60	22.00	28.50
17	266	147.92	19.03	25.27	29.84	215	155.30	18.97	24.45	28.50
18	215	151.37	19.07	23.84	29.81	266	155.18	18.92	24.55	28.44
19	546	145.02	20.04	22.60	29.64	569	155.26	18.91	24.15	28.44
20	571	154.10	19.50	20.77	29.61	269	157.13	18.50	24.76	28.40
21	569	157.98	18.97	20.90	29.60	571	151.54	19.45	23.22	28.35
22	514	151.77	19.49	21.55	29.59	51	155.06	18.80	24.06	28.35
23	269	156.07	18.59	22.61	29.59	239	155.75	18.60	24.43	28.30
24	531	151.51	19.69	20.96	29.56	514	151.13	19.44	23.18	28.28
25	239	153.57	18.69	22.99	29.54	531	149.61	19.65	22.74	28.24
26	51	158.15	18.83	20.62	29.46	524	152.56	19.01	23.51	28.17
27	516	150.40	19.57	21.19	29.45	546	147.20	19.99	22.29	28.16
28	524	153.04	19.07	21.66	29.44	544	149.77	19.50	22.70	28.16
29	544	152.89	19.54	20.36	29.44	512	154.69	18.58	24.03	28.15
30	212	150.11	18.09	25.41	29.43	212	157.80	17.97	25.11	28.13
Mean of sel.		153.14	19.51	22.47	29.93		154.15	19.40	24.09	28.64
Overall mean		143.45	18.43	20.82	28.06		143.45	18.43	20.82	26.63
Gain (%)		6.76	5.86	7.95	6.64		7.46	5.26	15.71	7.56

## Discussion

### Analysis of deviance

The LRT for the FSTME model revealed that the progeny and G×E interaction effects are significant (P < 0.01) for the DM, SW, and SY traits. Consequently, the respective variance components are significantly different from zero and so are the respective coefficients of determination (Table 1). The fit of the frequentist models was checked by AIC. This criterion indicated the full model as the most suitable to estimate the variance components and predict the genotypic values (Table 2). For the Bayesian approach, the full (considering genotype and G × E interaction effects) and reduced (only genotype or G x E interactions effects) models were compared through DIC (Deviance Information Criterion), which suggests that models with smaller DIC are better supported by the data. According to Spiegelhalter *et al*. [42], models with differences in DIC values lower than 2 need to be considered as equally well. Therefore, since DIC values obtained were higher than 2, it is possible to indicate the superiority of full model over the restrict models.

The generalization of AIC is the most common method of assessing the fit of a statistical model estimated via Bayesian inference (DIC). The effects of Bayesian models can be used as an inference to the test of hypothesis [19]. In Bayesian statistics, the lowest expected deviance has the HPD [60], and this was observed in the present study for the MTME model (Tables 2 and 3).

In both criteria (AIC and DIC) for the choice of statistical models, the obtained results revealed that the MTME models showed the best fit, explaining the genetic variability of the experiment and selection considering the genetic (progeny) and environmental interaction effects (Table 2).

### Variance components

Variance components are the variances associated with the random effects of a model. Knowing them is of great importance in genetics and breeding, since the population and the breeding method to be used depend on information that can be obtained from these components. The solution of mixed-model equations depends on knowledge of the variance and covariance matrix, whose structure is known, but its components often are not. At present, the standard method for the estimation of variance components is REML, developed by Patterson and Thompson [32].

The BLUP method [33] maximizes the correlation between the predicted and the true genotypic value; i.e., it minimizes the prediction error variance (PEV). Additionally, it is not biased, as we expect the predicted genotypic value to be equal to the true genotypic value [61]. Further, BLUP allows for the simultaneous use of several sources of information as well as information originating from experiments carried out in one or various locations and evaluated in one or various harvests [62].

Although the mixed-model methodology by the frequentist approach has several desirable characteristics [49], the adoption of Bayesian statistical inference for genetic evaluation in the breeding of crop species has shown to be advantageous. Bayesian models have been used since 1986 [63] and further exploited in recent years [23–25,64,65] due to the great computational advancements and new methodological applications and elucidations.

Bayesian analysis is based on the knowledge of the posterior distribution of the parameters to be estimated. This allows for the construction of exact credibility intervals for the estimates of random variables, variance components, and fixed effects [66]. Higher values for the interval with 95% credibility of distribution for the *broad-sense heritability* parameter found in this study (Table 4) were also presented in the study of Torres *et al*. [25] to estimate genetic parameters for N-uptake efficiency and N-utilization efficiency under contrasting N levels in the soil via BMTME models. The difference between mean, mode, and median of broad-sense heritability estimates (Table 4) reflects some lack of symmetry in the posterior distribution estimates [38]. However, for the SY trait, differences between the BSTME and BMTME are clear when we analyze the posterior densities, mainly because the posterior MTME resulted in a more narrow and symmetric distribution, confirming the increase in precision (Fig 3). When the prior distribution is informative, the credibility interval tends to be narrower than the confidence intervals. When the mixed-model parameters are assigned non-informative distributions, Bayesian and frequentist inferences should be equivalent [67].

Mathew *et al*. [68] showed that Bayesian inference is superior to frequentist inference when the posterior distribution of a variance component is bimodal. Mathew *et al*. [68], Waldmann *et al*. [69], Schenkel *et al*. [70], and Harville *et al*. [71] did not find relevant differences between the breeding values predicted by frequentist or Bayesian approach. Schenkel *et al*. [70] also observed that the breeding values presented the same bias and accuracy. Silva *et al*. [72] found results from noninformative analyses and results from REML/BLUP analyses (frequentist) for some components of variance and heritability and for breeding values. The specific results obtained by the frequentist and Bayesian approaches were similar (Table 3). This was expected, since non-informative prior distributions were used in Bayesian analysis. The modes of the marginal posterior distributions of the genetic parameters were similar to the corresponding REML estimates. From the Bayesian point-of-view, the estimates obtained via REML correspond to the modes of the combined posterior distributions of the variance components, obtained by Bayesian approach, given the use of uniform priors for the fixed effects and variance components [66].

The frequentist and Bayesian MTME models provided higher $\sigma_{g}^{2}$ estimates and lower $\sigma_{\mathrm{int}}^{2}$ estimates, which resulted in higher $h_{prog}^{2}$ for all evaluated traits. The highest $h_{prog}^{2}$ estimates were found for DM and SW and the lowest for SY, which confirms that DM and SW are less complex traits and are thus less influenced by the environment than SY [73–75].

The genotypic coefficient of variation (*CV*_*g*_) quantifies the magnitude of genetic variation available for selection, and thus high values are desirable [76]. In this way, the increase seen in this parameter with the use of the MTME models is important for breeding programs. The residual coefficient of variation (*CV*_*e*_) is a measurement of experimental precision of statistical and non-genetic nature. According to Resende and Duarte [52], *CV*_*e*_ is of moderate magnitude for the SY trait and low magnitude for the DM and SW traits, indicating good experimental precision. Moreover, as expected, there were no alterations in the *CV*_*e*_ estimates when the FSTME and FMTME models were used (Table 3).

### Genetic correlations

Studies on genotypic, phenotypic, and environmental correlations in soybean involve traits that are evaluated from flowering to maturity; notably, yield and its components [77–80]. Our results corroborate those reported by Cober *et al*. [81], who also obtained a high correlation between DM and SY and no linear associations between the DM-SW and SW-SY pairs. The authors argued that the genes controlling maturity in soybean have pleiotropic effects with grain yield. Ablett *et al*. [82] and Lee *et al*. [83] reported that late maturity was associated with high yield. Liu *et al*. [84] investigated the genetic architecture of three growth period traits and confirmed that the soybean growth stages are highly correlated with grain yield. Li *et al*. [85] and Zhang *et al*. [75] observed that the association between the BARC-016957-02165 marker and seed yield was located in the same region as a QTL controlling pod maturity on chromosome 6, which explains the high correlation observed between these traits, as the QTL were closer to each other.

According to Pollak *et al*. [14], selection biases may occur when traits are analyzed individually. This bias was observed in the present study, where the genetic correlation value between the DM and SY traits exceeded the parameter space (value higher than 1) (Table 5). Viana *et al*. [15] evaluated two traits in popcorn and also found that the genetic correlation obtained with the single-trait model exceeded the parameter space.

According to Thompson and Meyer [86], the increase in accuracy obtained with the use of multi-trait BLUP analysis compared with single-trait analysis is proportional to the difference between the genetic and environmental correlations of the analyzed traits. In the context of whole-genome prediction, Jia *et al*. [87], Guo *et al*. [88], and Jiang *et al*. [22] found that joint prediction of multiple traits benefits from genetic correlations between traits and significantly improves prediction accuracy compared to single-trait methods, specifically for low-heritability traits that are genetically correlated with a high-heritability trait. This fact was observed in our study, in which the DM variable showed high heritability and high correlation with SY, consequently generating significant increases in selection accuracy for SY. However, for both methodologies—frequentist and Bayesian—there was no significant increase in selection accuracy for the SW trait, as verified by its low correlation with the other evaluated traits.

### Progeny selection

The observed differences in the genotypic values predicted by the Bayesian and BLUP/REML procedures were small, leading to a slight alteration in the ranking of the progeny selected by both procedures. This finding was confirmed by Rank Spearman correlation (Table 6). However, compared with the FSTME and BSTME models, the respective MTME models showed higher genetic gains for DM and, especially, for SY. According to Resende *et al*. [66], these are the conditions for there to be correspondence between the frequentist and Bayesian methodologies for the fixed and random effect parameters: attribution of non-informative priori for the fixed effects, normal priori for the random effects, and normal likelihood for the observations vector. These promises were used in the present study, which explains the obtained results.

Despite the high agreement between the progeny selected for the DM and SW traits by both procedures, there was little agreement for the SY trait, which resulted in greater gains predicted from selection via MTME. Piepho *et al*. [3] and Piepho *et al*. [62] recommended the use of multiple-trait models to predict breeding values in annual crops, because this procedure has the best statistical properties and provides more-accurate results.

Resende *et al*. [19] and Okeke *et al*. [89] stated that one of the main advantages of using multivariate models is higher selection accuracy. Okeke *et al*. [89] also reported that multi-environment models were useful for understanding G×E interactions. Higher *Ac*_*prog*_ were observed when obtained via MTME for all traits, with SY standing out with 42.29 and 21.37% increases in the frequentist and Bayesian models, respectively. Greater accuracy and efficiency of multiple-trait models were also reported by Viana *et al*. [15] in selection among and within half-sib families.

However, it must be stressed that the BSTME model obtained superior accuracy in comparison with the FSTME model for DM and SY, despite the similar broad-sense heritability values. This is explained by the use of the estimator of selection accuracy. In this regard, Resende *et al*. [19] described that when Bayesian accuracy is higher than frequentist accuracy, the distribution of the parameters attributed to Bayesian approach were probably more adequate than those associated with the traditional model. The opposite can be considered true for the SW variable, for which the frequentist models obtained better results due to a better adjustment of the normal distribution of the parameters attributed to the data. These conclusions are also valid for the MTME models, which exhibited different obtained accuracies; however, for the SY variable, the FMTME model showed the best fit according to the mean accuracy of the progeny (Table 3).

As can be seen in Tables 7 and 8, desirable gains are obtained in selecting the best progeny for the DM, SW, and SY variables for all models based on the AGI. However, the high positive correlation between the DM and SY traits can favor the selection of high-yielding and late-cycle progeny. In this case, selection indices can help breeders select progeny that exhibit gains for both traits simultaneously [90]. Although similar gains were found for both approaches employed, it should be stressed that there was a slight increase in gain (%) when the 30 best progeny were selected for the DM and SY traits using Bayesian approach. This is a desirable factor that should be taken into account by breeders.

According to Silva *et al*. [1], individual plants or progeny in the *F*_2:3_ or *F*_2:4_ generations can be selected aiming at the adoption of the recurrent selection method described by Hallauer *et al*. [91], by exploiting genetic variability among and within progeny. Silva *et al*. [1] stated that the *F*_2:4_ generation is suitable for selection, since 87.5% (1.75) of the total additive genetic variance ($2\sigma_{a}^{2}$) that will be available in *F*_∞_ is already available in *F*_2:4_. Thus, progeny selection in *F*_2:4_ through a more precise method is relevant.

Early in the generation of the base populations of the soybean breeding programs, many populations are commonly obtained at the expense of the number of progeny to be evaluated; i.e., the evaluation of future lines, be them for high or low heritability, is based on samples with a finite (small) number of progeny. Thus, Bayes' theorem is recommended for those situations, as it gives precise solutions to the problem of finite-size samples, because for each data set—large or small—there is an exact posterior distribution to draw inferences.

The MTME models provided better results than the single-trait models using frequentist and Bayesian approach. Therefore, the former procedures can be efficiently applied in the genetic selection of segregating soybean progeny. However, it is necessary to use an adequate statistical tool that provides algorithms and routines to efficiently perform the analyses. Though not necessarily easy, the use of Bayesian inference in quantitative genetics in the breeding of crop species [69,72] is a tendency in breeding programs [5].

## Conclusion remarks

For our data set, the average BMTME processing time using an Intel(R) i7-5500U (2.4 GHz) processor with 8 GB of RAM was 1 h 40 min and 35 s, corresponding to approximately 0.006 s for each MCMC iteration. Silva *et al*. [72] considered this performance plausible, but pointed out that improvements can be obtained using the conditional decompositions proposed by Hallander *et al*. [92]. For the same purpose, in addition to improving the prior information, Montesinos-López *et al*. [13] proposed a Bayesian model for analyzing multiple traits and multiple environments for the whole-genome prediction model. The authors also developed an R-software package that offers specialized and optimized routines to efficiently perform the analyses under the proposed model. By contrast, the FMTME model took approximately 14 s to converge. Despite the considerable difference in processing time of the analysis and output size of the results (around 1.03 GB) due to the high number of interactions adopted, the Bayesian model showed to be efficient for the proposed objective. Furthermore, it provided additional results to those obtained by the frequentist approach, with noteworthy credibility intervals.

The Bayesian models have desirable potentials when using informative prior distributions, providing parameters with lower standard deviations and/or possible genetic gains. However, the quality of the informative prior may have questionable origins and may not generate considerable advantages. Silva *et al*. [72] showed it can be advantageous to implement a Bayesian framework for mixed-model analysis in the breeding of crop species using informative priors. However, for potential future studies in plant breeding, the implementation of informative prior fitted to MTME models can be the next step to be assessed.

## Supporting information

S1 Table Data set necessary to replicate the findings of our research. (TXT)

S2 Table Scripts to run the multi-trait multi-environment models. Data set necessary to replicate the findings of our research.
(TXT)

S1 Fig On the diagonal, posterior density of 95% intervals of the estimate of variance components via the Bayesian multi-trait multi-environment (BMTME) model; off the diagonal the traits’ correlations by score for number of days to maturity (DM), 100-seed weight (SW; grams), and average seed yield per plot (SY; grams). (TIFF)

S2 Fig Posterior density for the Bayesian single-trait multi-environment model (BSTME) of the estimate of variance components for maturity (DM). The solid color represents the posterior distributions of 95% intervals and the solid vertical line indicates the mean for number of days.
(TIFF)

S3 Fig Posterior density for the Bayesian single-trait multi-environment model (BSTME) of the estimate of variance components for 100-seed weight (SW). The solid color represents the posterior distributions of 95% intervals and the solid vertical line indicates the mean.
(TIFF)

S4 Fig Posterior density for the Bayesian single-trait multi-environment model (BSTME) of the estimate of variance components for average seed yield per plot (SY). The solid color represents the posterior distributions of 95% intervals and the solid vertical line indicates the mean.
(TIFF)
