# Supplementary figures and images for: Multi-trait multi-environment models in the genetic selection of segregating soybean progeny
Source: PLoS One. 2019 Apr 18;14(4):e0215315. doi: 10.1371/journal.pone.0215315 (PMC6472761; doi:10.1371/journal.pone.0215315)

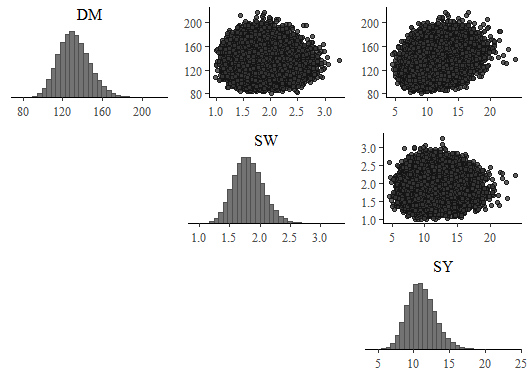

Supplement: S1 Fig — (TIFF) [file pone.0215315.s003.tiff]

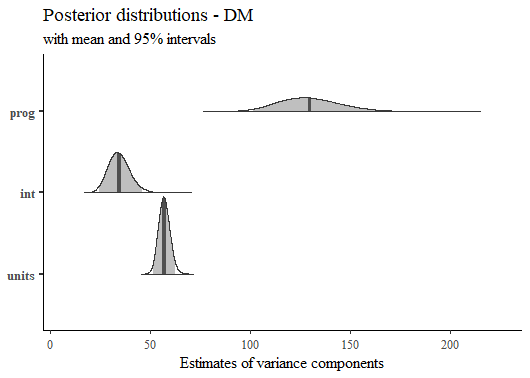

Supplement: S2 Fig — The solid color represents the posterior distributions of 95% intervals and the solid vertical line indicates the mean for number of days. (TIFF) [file pone.0215315.s004.tiff]

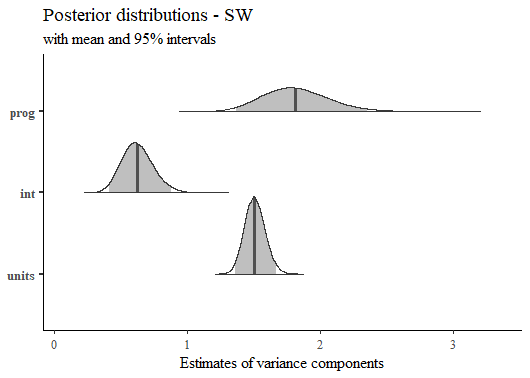

Supplement: S3 Fig — The solid color represents the posterior distributions of 95% intervals and the solid vertical line indicates the mean. (TIFF) [file pone.0215315.s005.tiff]

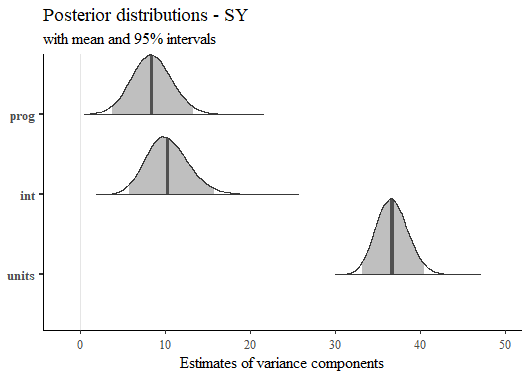

Supplement: S4 Fig — The solid color represents the posterior distributions of 95% intervals and the solid vertical line indicates the mean. (TIFF) [file pone.0215315.s006.tiff]
